# Functional Investigation and Two-sample Mendelian Randomization Study of Inguinal Hernia Hub Genes Obtained by Bioinformatics Analysis

**DOI:** 10.2174/0115734099282407240325054745

**Published:** 2024-04-05

**Authors:** De Kun Lu, Zheng Chang Guo, Jia Jia Zhang, Xin Yu, Zong Yao Zhang

**Affiliations:** 1 Department of General Surgery, The First Affiliated Hospital of Anhui University of Science and Technology, No.203 Huai Bin Road, Tian Jia'an District, Huainan, 232007, China;; 2 Department of General Surgery, Zhenjiang First People's Hospital, No.8 Electricity Road, Run Zhou District, Zhenjiang, China;; 3 Department of General Surgery, The First Affiliated Hospital of Anhui Medical University, No. 218 Jixi Road, 230022, Hefei, China

**Keywords:** Inguinal hernia, text mining, gene, bioinformatics, mendelian randomization, drug discovery

## Abstract

**Background:**

Inguinal hernia in adults is a common and frequent disease in surgery, prone to occur in the elderly or in those with a weak abdominal wall. Despite its prevalence, Molecular mechanisms underlying inguinal hernia formation are unclear.

**Objectives:**

This study aims to identify potential gene markers for inguinal hernia and available drugs.

**Methods:**

Pubmed2Ensembl text mining was used to identify genes related to “inguinal hernia” keywords. The GeneCodis system was used to specify GO biological process terms and KEGG pathways defined in the Kyoto Encyclopedia of Genes and Genomes (KEGG). The STRING tool was used to construct protein-protein interaction networks, which were then visualized using Cytoscape.CytoHubba and Molecular Complex Detection were utilized to analyze the module (MCODE). A GO and KEGG analysis of gene modules was conducted using the DAVID platform database. Hub genes are those that are concentrated in prominent modules. The drug-gene interaction database was also used to identify potential drugs for inguinal hernia patients based on their interactions between the hub genes. Finally, a Mendelian randomization study was conducted based on genome-wide association studies to determine whether hub genes cause inguinal hernias.

**Results:**

The identification of 96 genes associated with inguinal hernia was carried out using text mining techniques. It was constructed using PPI networks with 80 nodes and 476 edges, and the sequence of the genes was performed using CytoHubba. MCODE analysis identified three gene modules. Three modules contain 37 genes clustered as hub candidate genes associated with inguinal hernia patients. The PI3K-Akt, MAPK, AGE-RAGE, and HIF-1 pathways were found to be enriched in signaling pathways. Sixteen of the 37 genes were found to be targetable by 30 existing drugs. The relationship between hub genes and inguinal hernia was examined using Mendelian randomization. The research revealed nine genes that may be connected with inguinal hernia, such as POMC, CD40LG, TFRC, VWF, LOX, IGF2, BRCA1, TNF, and HGF in the plasma. By inverse variance weighting, ALB was associated with an increased risk of inguinal hernia with an OR of 1.203 (OR [95%] = 1,04 [1.012 to 1.089], *p* = 0.008).

**Conclusion:**

We identified potential hub genes for inguinal hernia, predicted potential drugs for inguinal hernia, and reverse-validated potential genes by Mendelian randomization. This may provide further insights into asymptomatic pre-diagnostic methods and contribute to studies to understand the molecular mechanisms of risk genes associated with inguinal hernia.

## 1 INTRODUCTION

Inguinal hernia is a prevalent condition in surgery, particularly in adults. Globally, hernia repairs are the most frequently performed general surgical procedure [1]. Inguinal hernia arises when intra-abdominal organs protrude through an inguinal defect [2]. The hernia mass typically protrudes when standing or when there is an increase in intra-abdominal pressure and disappears entirely when lying down. There are two types of inguinal hernia based on their location. Hiatal hernias develop in the inner ring of the inguinal canal, protrude lateral to the inferior abdominal wall artery and account for more than 90% of all inguinal hernias [3]. Normally, a straight hernia protrudes from the body from the triangle, with the hernia ring located medial to the inferior abdominal wall artery [4].

The pathogenesis of inguinal hernia in adults is primarily focused on clinical aspects such as increased intra-abdominal pressure and abdominal wall weakness [5]. However, it is not clear whether genetic and molecular alterations contribute to the development of inguinal hernias. Inguinal hernia can occur at any age, and abnormal composition and structural disorders of the extracellular matrix in connective tissue have been found to play a vital role in developing inguinal hernia in children [6], leading to hernia. The development of inguinal hernia is closely related to histopathological morphological changes and changes in cellular biology and function [7], suggesting that genomics plays a crucial role in hernia disease.

According to this study, there will be a combination of text mining, biological process analysis, pathway analysis, and protein- protein interactions that will be used in order to identify potential genes and signaling pathways closely associated with inguinal hernia. We seek to uncover the hidden molecular mechanisms associated with inguinal hernia. In our investigation, we pinpointed hub genes serving as prospective markers potentially instrumental for the diagnostic elucidation of inguinal hernia, and for advancing comprehension of the inherent drug targets affiliated with these hub genes.Subsequently, a Mendelian randomization study was conducted to probe the causal nexus between hub genes and inguinal hernia.

## 2 MATERIALS AND METHODS

### 2.1 Text Mining

Utilizing Pubmed2Ensembl, a BioMart extension that bridges over 2 million PubMed articles to approximately 100,000 Ensembl genes, we engaged in text-mining endeavors [8]. For this study, the term “inguinal hernia” was searched using “homo sapiens” as the species. The search parameters included “Ensembl Gene ID” and “Associated Gene Name” under GENE. The search was refined using “search for Pubmed IDs” and “filter on Entrez: PMID” to retrieve genes associated with inguinal hernia using text mining.

### 2.2 Analysis of Biological Processes and Pathways Enriched by Text Mining

The Genecodis web application is a powerful tool for interpreting genomic experiment results functionally [9]. By incorporating multiple information sources, it searches for coexisting gene annotations and ranks them according to their statistical significance. The GO biological process analogy was used to input and analyze our text-mining genes. As a result, significant enrichment genes in biological processes were selected. Genes enriched in KEGG pathways were further analyzed using KEGG pathway annotations. Significantly enriched pathways were then further examined using genes involved in those pathways [10]. Our objective was to identify the signaling pathways of biological processes highly relevant to the pathophysiology of inguinal hernia.

### 2.3 Identifying Hub Genes by Integrating Protein-protein Interactions (PPI) Networks

Utilizing STRING, a repository encompassing roughly 24.6 million proteins derived from 5,090 organisms, is at our disposal. We conducted a Protein-Protein Interaction analysis predicated on the outcomes of the antecedent gene enrichment analysis. PubMed text mining data can be analyzed using STRING and integrated with multiple databases [11]. Over 3.1 billion interactions are included in this database, which can be used to analyze the relationship and interaction between proteins. Leveraging STRING, we constructed the Protein-Protein Interaction network of diverse genes.

Additionally, CytoHubba within Cytoscape was employed to discern hub genes. CytoHubba is a plugin for Cytoscape that provides eleven topological analysis methods for categorizing network nodes [12]. We screened out genes with a degree of 10 as determined by the correlation analysis.

### 2.4 Analysis of Subnetworks based on Molecular Complex Detection (MCODE)

The visualization network of molecular interactions was screened using MCODE, constructed by Cytoscape [13]. The network was visualized using Cytoscape, and MCODE was applied to the network to identify gene modules. Gene modules were sorted based on the network score. Modules with the highest scores were considered to be the most critical and representative. Among the 40 genes comprising the PPI network, additional investigation and validation were conducted on the two gene modules with the highest network scores.

### 2.5 Analysis of GO and KEGG Pathway Enrichment in Important Modules

Gene function analysis was executed employing the online DAVID tool to elucidate the functional attributes and potential biological implications of the identified genes [14]. A DAVID database is an integrated biological information discovery tool that gives users access to annotations, visualizations, and integrated discovery tools. It associates genes in the input list with biological annotation terms and identifies significantly enriched annotations statistically. Gene differentials are analyzed primarily for function and pathway enrichment. In the DAVID analysis, we set the cut-off point (FDR<0.05). There are three main categories of functional classification terms in the GO database: biological process, cellular component, and molecular function [15]. The KEGG tool is a knowledge base that analyzes, annotates, and visualizes gene functions systematically (www.genome.jp/kegg) [16].

### 2.6 Analysis of Drug-gene Interactions in Hub Genes

The Drug-Gene Interaction Database (DGIDB) is a useful tool for formulating hypotheses regarding targeted therapies or drug development priorities for mutated genes [17]. The database provides comprehensive information on gene interactions and drug interactions. In this study, we explored potential drug targets associated with the genes identified using DGIDB, with the aim of identifying existing drugs or compounds that could be used for treatment. By analyzing the information in DGIDB, we were able to identify potential drug targets and generate hypotheses about drug-gene interactions that could be further explored in future studies.

### 2.7 Mendelian Randomization Analysis

In recent years, Mendelian Randomization (MR) has ascended as a reliable methodology for deducing potential causal associations by leveraging Single Nucleotide Polymorphisms (SNPs) as Instrumental Variables (IVs), thereby evaluating the causal linkage between exposures and outcomes [18]. MR leverages genetic variations that exhibit robust associations with exposure factors as instrumental variables to deduce causal effects between exposure factors and study outcomes [19]. Based on the results of the CytoHubba analysis, 37 node genes (node degree of 10) were selected. Hub genes were also pinpointed within integrating protein-protein interaction networks, signifying the genes with the highest correlation degree in the module.; In addition, we selected the ALB gene, which has the highest association among the selected hub genes, to be expressed in the blood as an exposure factor to verify whether there is a correlation with inguinal hernia.To estimate the causal effect between hub genes and inguinal hernia, a two-sample MR analysis was conducted using the TwoSampleMR R package (v0.5.7) [20]. Independent SNPs were obtained using clumping, and those in linkage disequilibrium (LD) with r^2^ < 0.001 were removed. Traits were assessed at a genome-wideel (*p* < 5 ×10^−6^), while evaluating the instrumental strength of each SNP with the F statistic =(β/SE)^2^ [21]. The article presents the average F statistic of the SNPs used as instruments, an indicator of strong instruments when exceeding [22]. The Wald ratio test was utilized for single instruments, consisting of dividing the SNP exposure by the SNP outcome to obtain estimates [23]. For multiple instruments, the Inverse Variance Weighting (IVW) technique was applied, incorporating data from all instruments. The investigation into the causal links between key hub genes of interest and inguinal hernia within this Mendelian Randomization (MR) analysis primarily employed the standard Inverse Variance Weighted (IVW) approach. The IVW method is known for delivering reliable causal effect estimates of the exposure when the instrumental variables meet three core assumptions, positioning it as the most robust MR technique available. A causal relationship was established as statistically significant when the FDR-adjusted *p*-value was below 0.05 [24-26]. Heterogeneity was calculated for IVW estimates to display high variance across instruments, indicating the possibility of the presence of invalid instruments [27, 28]. We created a scatter plot to visualize the effects of SNPs on exposure against their effect on the outcome of MR results [29]. We utilized a forest plot to demonstrate the estimates of multiple instruments. A funnel plot was used to evaluate heterogeneity objectively, while a leave-one-out plot visualized MR estimates when a specific instrument was removed [30]. We included all cis-acting factors to ensure comprehensiveness, with technical abbreviations explained at their first occurrence for clarity. All analyses were conducted using R statistics (v4.3.0), and Fig. graphs were produced using the R software packages NMF (v0.23.0) and ggplot2 (v3.1.5).

## 3 STATISTICAL ANALYSIS

### 3.1 Data Source

Publicly available databases, such as GWAS catalog IEU openGWAS, were queried to locate eligible datasets inclusive of exposure and outcome [31, 32]. No additional ethical approvals were necessary since available databases were utilized. To reduce biased estimates due to possible confounding, the genetic backgrounds of the population in the MR study were restricted to individuals of a European group. Exposure data on hub genes' cis-expression quantitative trait loci (eQTLs) of individuals of European descent were extracted from publicly available genome-wide association study (GWAS) datasets [33]. If the gene was within 1 Mb of the SNP, it was considered a cis-eQTL and significant at a p-value below 5x10^-6^. Outcome data were obtained from the UK Biobank Consortium [34] and the Finnegan Consortium [35] associated with the IEU. Diagnostic data for inguinal hernia were extracted from the UK Biobank using International Classification of Diseases-10 codes and self-reported information [36]. The International Classification of Diseases-10 codes codes (ICD-10) for inguinal hernia were K40, K40.2, and K40.9, main inguinal hernia, denoting bilateral inguinal hernia (without obstruction or gangrene), unilateral or unspecified inguinal hernia (without obstruction or gangrene). To obtain more outcome information related to inguinal hernia, we utilized the phenotype codes K11-hering in the Finngen consortium [37, 38].

## 4 RESULTS

### 4.1 The Acquisition of Genes for Text Mining

**Fig. (1):**
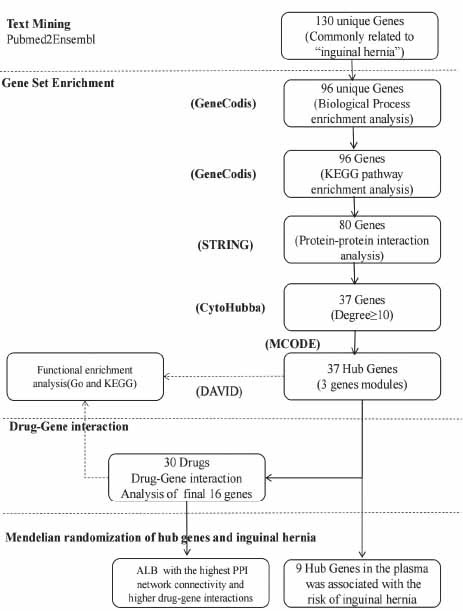
Summary of the whole study design. Text mining procedures were employed, utilizing Pubmed2Ensembl, to identify genes associated with inguinal hernia. GeneCodis was used for gene collection enrichment, specifically to identify genes enriched in GO biological process terms and KEGG pathways. STRING and CytoHubba were utilized to construct a protein-protein interaction network and to screen the proteins encoded by the hub genes based on node degrees. MCODE was applied to identify related protein network modules and calculate the score for each module. DAVID and ClueGO were employed to analyze GO biological process terms and KEGG pathways, providing insights into gene function and pathway associations. DGIDB was used to classify potential drug targets based on lists of significant genes, potentially identifying genes relevant to therapeutic interventions. MR analysis of hub genes and inguinal hernia.

A total of 130 genes were identified as associated with inguinal hernia by text mining as outlined in Materials and Methods (Fig. **1**). In order to ensure the accuracy and reliability of the analysis, all duplicate genes were removed, resulting in a final set of 96 genes that were deemed to be involved in the biological mechanisms underlying inguinal hernia in patients.

### 4.2 Analysis of Gene Ontologies and KEGG Pathways

Inguinal hernia patients were analyzed using GeneCodis to visualize GO functional and KEGG pathways and identify the most enriched terms. As a consequence of our analysis, annotations of 1634 biological processes were significantly enriched in 96 genes encoding 96 unique genes. The analysis revealed significant enrichment of these genes in five distinct categories: “positive regulation of gene expression” (*p* = 2.19E-10), “negative regulation of apoptotic process” (*p* = 2.56E-09), “response to activity” (*p* = 3.20E−09), “negative regulation of cell population proliferation” (*p* = 2.84E−09), and “positive regulation of miRNA transcription” (*p* = 4.44 E−09). These categories involved 18, 17, 7, 16, and 7 genes, respectively, as demonstrated in Table **1**.

In addition, other biological processes were highly enriched “positive regulation of peptidyl-tyrosine phosphorylation”, “positive regulation of transcription, DNA-templated”, “response to xenobiotic stimulus”, “positive regulation of phosphatidylinositol 3-kinase signaling”, and “regulation of gene expression”. GeneCodis was used to conduct KEGG pathway enrichment analysis on the identified text-mining genes in order to evaluate their functions and signaling pathways. Based on the KEGG pathway enrichment analysis, 96 text-mining genes belong to 218 of the most important pathways. As demonstrated in Table **2**, the major signaling pathways enriched were “Malaria” (*p* = 3.38E−07), “EGFR tyrosine kinase inhibitor resistance” (*p* = 6.07E−07), “HIF-1 ” (*p* = 6.67E−07), “Focal adhesion” (*p* = 1.55E−05), and “African trypanosomiasis” (*p* = 1.87E−05) involving 7, 8, 9, 10, and 5 text mining genes, respectively (Table **2**).

Other highly concentrated pathways included “Cytokine-cytokine receptor interaction,” “Proteoglycans in cancer,” “PI3K-Akt signaling pathway,” “Pathways in cancer,” and “Renal cell carcinoma.”

### 4.3 Construction of PPI Networks, Identification of Hub Genes, and Modular Analysis

**Fig. (2):**
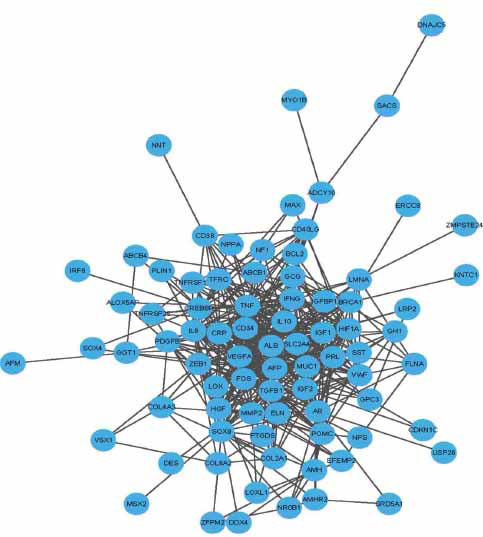
The Protein-Protein Interaction (PPI) network of the 96 target genes was visualized using Cytoscape. Sixteen of these genes were excluded from the constructed PPI network.CytoHubba, a component of Cystoscope, was executed. A topological network algorithm was employed to assign a value to each gene and rank them based on their degree of correlation analysis. The darker the pigment, the higher the score, indicating greater significance of the gene (Fig. **3A**). On the basis of the results of the analysis, 37 nodal genes with a degree of 10 were selected, as demonstrated in Table **3**.

In this study, The PPI network of 96 target genes was established through the STRING website, comprising 80 nodes and 476 edges. A total of sixteen genes were not included in the PPI network. The CytoHubba component for Cystoscope assigned a value to each gene based on a topological network algorithm and ranked each gene based on its correlation analysis degree. Based on the results of the analysis, 37 nodal genes (nodal degree≥10) were chosen as hub genes. Hub genes are also identified in the PPI network, referring to genes with the highest connection in the module. ALB, TNF, IL6, VEGFA, IGF1, HIF1A, FOS, MMP2, CRP, and IL10 were the ten hub genes with the highest connectivity in the module. Further analysis involved using the MCODE plugin to obtain more gene modules, resulting in three modules. We conducted further analysis on the two highest scores modules containing a total of 34 genes. Module 1 consists of 18 nodes and 140 edges, while Module 2 comprises 10 nodes and 22 edges. On the STRING website, the target genes are submitted. A Protein-Protein Interaction (PPI) network consisting of 96 target genes was established through the analysis. The constructed PPI network had eighty nodes and four hundred seventy-six edges (Fig. **2**).

**Fig. (3):**
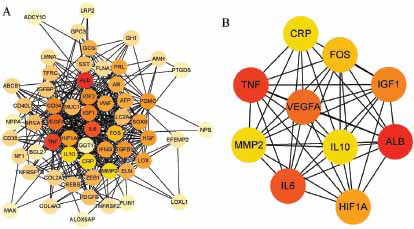
(**A**) Categorize the degree and analyze Hub genes. All nodes of PPI were presented according to degree by CytoHubba. The degree decreases from inside to outside, and the color changes from dark to light. (**B**) The first 10 hub genes in the macro module were identified by CytoHubba plug-in. The image shows the degree from red to yellow, and the significance of genes declines.

The center genes in the PPI network, or the gene with the most connections in the module, were then identified, as demonstrated in Fig. (**3A**), ALB, TNF, IL6, VEGFA, IGF1, HIF1A, FOS, MMP2, CRP, and IL10 were the 10 hub genes with the highest connectivity in the module (Fig. **3B**).

**Fig. (4):**
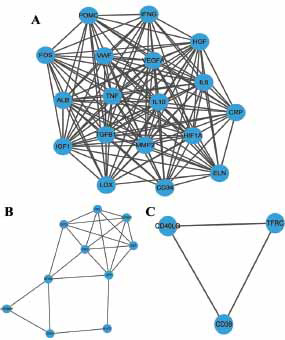
(**A**). Description and enrichment analysis of the 37 nodal hub genes (nodal degree ≥ 10), (**A**) Three modules were extracted from the Protein-Protein Interaction (PPI) network using the MCODE algorithm. (**B**). Module 2, the most significant module, consists of 10 nodes. (**C**). Module 3, another highly significant module, comprises 3 nodes.

To obtain additional gene modules, we analyzed the 37 previously identified hub genes with the MCODE component to obtain 3 modules (Figs. **4A**-**C**). Calculating network scores allowed us to identify the two modules (consisting of 34 genes) with the highest scores for further analysis. Module 1 contained 18 nodes and 140 edges. The second module contained ten nodes and twenty-two edges.

**Fig. (5):**
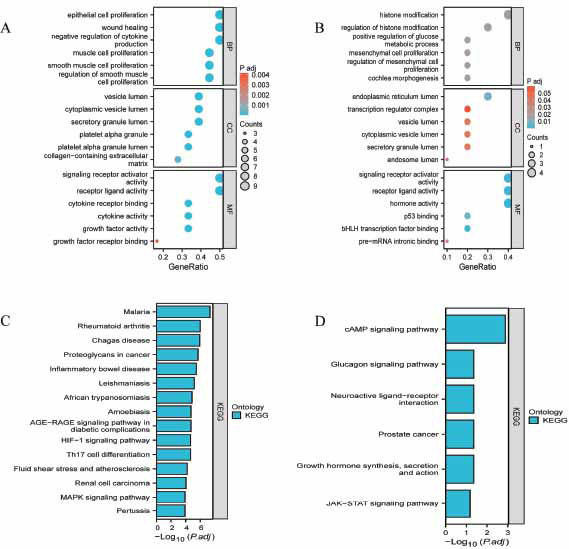
(**A**) Gene ontology and KEGG pathway analysis of the genes in the first two modules. Top 18 significantly enriched GO terms in module 1. Fig. (**B**) Top 10 Significantly enriched GO terms in module 2. Fig. (**C**) Top 18 significantly enriched KEGG pathways in module 1. Fig. (**D**) Top 10 significantly enriched KEGG pathways in module 2. The functional and pathway enrichment analyses were performed using DAVID.KEGG: Kyoto Encyclopedia of Genes and Genomes.

To delve deeper into the enrichment of hub genes, Gene Ontology and KEGG pathway analyses were carried out on the two modules selected in the previous phase. The results indicated that the majority of the 18 genes in module 1 were associated with the modulation of smooth muscle cell proliferation (BP), the vesicle lumen (CC), and signaling receptor activator activity (MF) (Fig. **5A**). The 10 genes in module 2 were primarily related to histone modification (BP), endoplasmic reticulum lumen (CC), and signaling receptor activator (MF) (Fig. **5B**). Pathway enrichment analysis revealed that the genes in module 1 were associated with the AGE-RAGE, HIF-1, and MAPK signaling pathways (Fig. **5C**). In contrast, module 2 genes were correlated significantly with the cAMP signaling pathway, the glucagon signaling pathway, and the JAKSTAT signaling pathway (Fig. **5D**).

### 4.4 Drug-gene Interaction Analysis of Hub Genes

The final confirmed genes were used to analyze drug-gene interactions, and a preliminary list of 30 compounds was compiled (Table **4**).

Thirty medications targeting 16 of the 18 hub genes (excluding POMC and LOX) have the potential drug target to treat inguinal hernia. The 18 hub genes most significantly enriched in the HIF-1 signaling Pathway, Malaria, cancer, and African trypanosomiasis pathways. The primary connections between pharmaceuticals, genes, and pathways are depicted in Fig. (**6**).

### 4.5 MR Analysis of Hub Genes and Inguinal Hernia

The relationship between hub genes and inguinal hernia was examined using Mendelian randomization. The research revealed nine genes that may be connected with inguinal hernia, such as Prochorionic Villous Corticotropin (POMC), CD40 Ligand (CD40LG), Transferrin Receptor (TFRC), Von Willebrand Factor (VWF), Lysosomal Oxidase (LOX), Insulin-like Growth Factor 2 (IGF2), DNA repair-related BRCA1 (BRCA1), Tumor Necrosis Factor (TNF), and Hepatocyte Growth Factor (HGF) in the plasma.

**Fig. (7):**
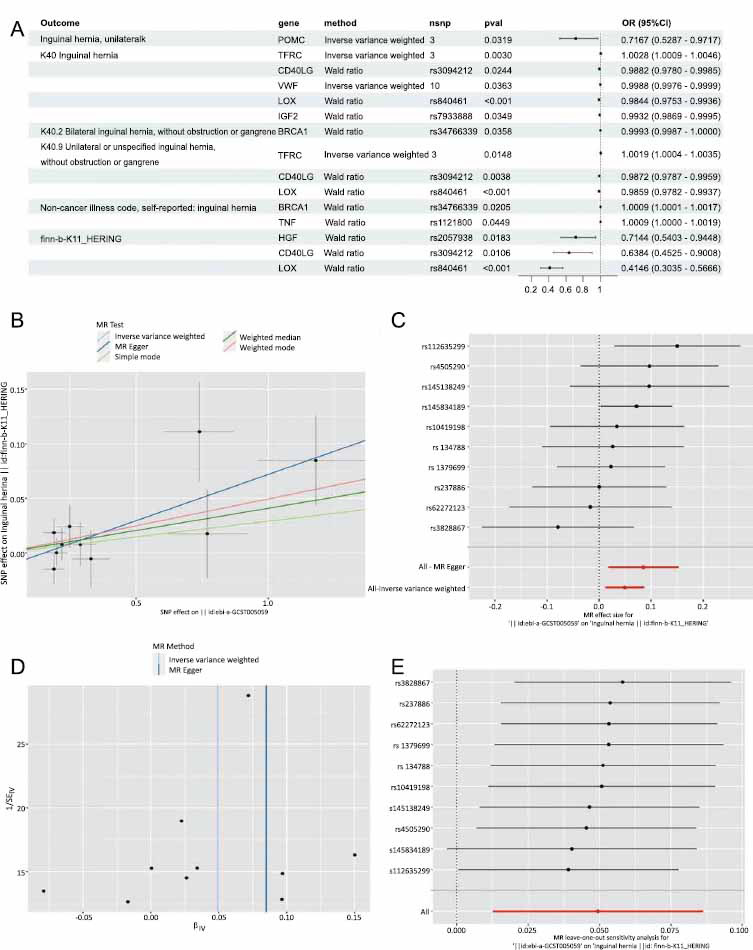
**(A)** Mendelian randomization study results. Forest plot showing the causal effect of hub genes on the risk of inguinal hernia. (**B**) Scatter plot showing the causal effect of ALB on the risk of inguinal hernia. (**C**) Forest plot showing the causal effect of each SNP on the risk of inguinal hernia. (**D**) Funnel plots to visualize overall heterogeneity of MR estimates for the effect of ALB on inguinal hernia. (**E**) Leave-one-out plot to visualize the causal effect of ALB on inguinal hernia risk when leaving one SNP out.

The IVW method indicates that genetically-predicted POMC (OR [95%]=0.716 [0.5287 to 0.97^17^], *p* = 0.0319) and VWF (OR [95%]=0.9988 [0.9976 to 0.9999], *p* = 0.0363) possess a clear protective effect on unilateral inguinal hernia. In addition, the Wald Ratio method shows that HGF (OR [95%]=0.7144 [0.5403 to 0.9448], *p* = 0.0183), CD40LG (OR [95%]=0.6384 [0.4525 to 0.900^8^], *p* = 0.0106), IGF2 (OR [95%]=0.9932 [0.9869 to 0.9995], *p* = 0.0349), BRCA1 (OR [95%]=0.9993 [0.9987 to 1.0], *p* = 0.0358), and LOX (OR [95%]=0.4146 [0.3035 to 0.56^66^], *p* < 0.001) may also significantly protect against inguinal hernia (Fig. **7A**). Besides, both TFRC and TNF could significantly increase the risk of inguinal hernia (TFRC: IVW: OR [95%]=1.0028 [1.0009 to 1.00^46^], *p* = 0.003; TNF: Wald ratio: OR [95%]=1.0009 [1.0 to 1.00^19^], *p* = 0.04).

We subsequently identified the ALB gene exhibiting the highest connectivity within the PPI network and possessing higher interaction scores in drug-gene interactions. The correlation between the ALB gene and inguinal hernia was subsequently validated through a two-sample Mende randomization. Table **S1** presents the SNP characteristics related to ALB and inguinal hernia. All SNPs were not weak instrumental variables. The causal effects of each genetic variation on inguinal hernia are shown in Fig. (**7B**). We assessed the causal association between ALB levels and inguinal hernia. Using the IVW method, we found that ALB was associated with the risk of inguinal hernia with an OR of (OR [95%]=1,05[1.012 to 1.089], *p* = 0.008). The MR-Egger method showed significant statistical significance (OR [95%]=1.088 [1.018 to 1.163], *p* = 0.03). The results of simple mode (*p* = 0.5), weighted median (*p* = 0.13), and weighted mode (*p* = 0.193) did not reach statistical significance. Cochrane’s Q and MR Egger regression equation were used to evaluate the level of heterogeneity and horizontal pleiotropy. The causal effect demonstrated by the funnel plot appeared to be roughly symmetrical (Fig. **7C**). Moreover, the MR Egger regression revealed that the intercept did not show evidence of horizontal pleiotropy (*p* = 0.528), providing further assurance that pleiotropy did not bias the causal effect.

Additionally, as illustrated in Fig. (**7D**), we systematically conducted MR analysis once more after removing each SNP. The results remained consistent, suggesting that the causality observed was significant for all SNPs. This implies that there was no dominant SNP influencing ALB levels and inguinal hernia, confirming the validity of the previous MR results.

## 5 DISCUSSION

By employing a series of bioinformatics techniques to investigate gene expression, we were able to identify 130 genes associated with inguinal hernia patients. Among the enriched genomes are biological process terms, which may be associated with inguinal hernia including positive regulation of gene expression, negative regulation of apoptotic process, response to activity, negative regulation of cell population proliferation, positive regulation of miRNA transcription, positive regulation of peptidyl-tyrosine phosphorylation, positive regulation of transcription, DNA-templated, response to xenobiotic stimulus, positive regulation of phosphatidylinositol 3-kinase signaling and regulation of gene expression. In addition, KEGG pathway analysis results for 130 genes associated with inguinal hernia, such as Malaria, EGFR tyrosine kinase inhibitor resistance, HIF-1 signaling pathway, Focal adhesion, African trypanosomiasis, Cytokine-cytokine receptor interaction, Proteoglycans in cancer, PI3K-Akt signaling pathway, and Pathways in cancer. Cancerous pathways may be associated with inguinal hernia, but this correlation must be further investigated. The PPI network and CytoHubba analyzed the correlation connectivity between these genes (Fig. **3A**) and identified the top ten hub genes, including ALB, TNF, IL6, VEGFA, IGF1, HIF1, MMP2, CRP, and IL10 (Fig. **3B**). Nevertheless, these alleles may also have a significant impact on the development of inguinal hernia. The MCODE analysis generated 37 hub genes, including ALB, TNF, IL6, VEGFA, IGF1, HIF1A, FOS, CRP, IL10, MMP2, CD34, IGF2, SOX9, POMC, TGFB1, AR, HGF, IFNG, LOX, VWF, ZEB1, AFP, PRL, ELN, GCG, BRCA1, CREBBP, MUC1, PDGFB, SLC2A4, SST, CD40LG, TFRC, IGFBP1, AMH, CD38, COL2A1 that participated in the HIF-1, PI3K-Akt, MAPK, AGE-RAGE signaling pathway in diabetic complications.

Interleukin 6 (IL-6) is indeed an autocrine growth factor produced by mesangial cells and has been linked to their pathological proliferation [39]. The OSM and LIF genes, members of the IL-6-related family, are pleiotropic cytokines with similar genetic structures and physiological functions that play important roles in chronic inflammation, autoimmunity, infectious diseases, and cancer [40-43]. LIF induces the synthesis of acute-phase proteins and influences the recruitment of cells into inflammatory regions [44]. OSM secreted by macrophages in combination with IL-1β promotes the production of MMP-1 in human chondrocytes and synovial fibroblasts, which in turn causes collagen destruction in cartilage [45]. The above findings suggest that immunological factors may be an important factor in the development of abnormal collagen metabolism and herniation. Insulin-like growth factor-1 receptor (IGF1) gene haploinsufficiency on chromosome 15q26.3 is linked to reduced prenatal and postnatal growth [46], developmental delay [47], dysmorphic features [48], and skeletal abnormalities [49]. In addition to being associated with diaphragmatic hernia and renal anomalies, more proximal deletions of chromosome 15q26 may be associated with diaphragmatic hernia. IGF1 has the function of regulating the synthesis and degradation of extracellular matrix, stimulating the proliferation of fibroblasts and smooth muscle cells, collagen deposition and fibrous connective tissue proliferation [50]. *In vitro*, the accelerated division and proliferation of fibroblasts and smooth muscle cells cultured with IGF-1 [51] and the increase in cell size may be one of the factors contributing to the formation of hernia. Matrix metalloproteinases (MMPs) are a family of zinc-dependent peptide endonucleases that are the main enzymes involved in the degradation of collagen, including MMP-1, MMP-2, MMP-9 and others [52-54]. During collagen degradation, collagenases unscrew the collagen triple helix structure and cleave it into quarter- and three-quarter-length fragments, which are then further degraded by gelatinases [55]. MMP-2 was found to be abnormally high at both mRNA and protein levels in serum and fascial tissues of patients with ventral wall hernias [56]; some studies showed that MMP-9 expression was increased in the hernia-positive group [57]. The results of numerous studies have shown that a decreased ratio of type I to type III collagen is common in the fascial tissue of patients with abdominal wall hernias, confirming that collagen abnormalities contribute to the development of abdominal wall hernias [58]. HIF-1 signaling pathway is involved in hypoxia adaptation, inflammation and tumor growth. The IL6 family gene OSM of the HIF pathway, together with IL-1β, promotes the production of MMP-1 in human chondrocytes and synovial fibroblasts [59, 60], resulting in cartilage collagen destruction, and the pro-inflammatory factor IL-17 released by Th17 cells in the HIF-1 pathway also promotes inflammatory cell infiltration and cartilage collagen destruction [61]. These findings suggest that HIF-1 induces inguinal hernia through abnormalities in collagen metabolism, possibly *via* immunoinflammatory factors. The PI3K-Akt signaling pathway mainly regulates cell proliferation, migration and angiogenesis [62-64]. Activation of PI3K phosphorylates Akt to form p-Akt, which further phosphorylates the downstream complex and tryptophan residues, thereby activating downstream factors such as VEGF and MMPs to perform their functions of promoting angiogenesis, cell proliferation and migration [65]. SRC is an important member of the Src family of kinases (SFKs) and is required for TGF-β stimulation of type I collagen secretion. In addition, mouse models with abnormal TGF-β signaling molecules cause severe abdominal wall defects [66, 67]. Thus, SRC mutations may lead to abnormal type I collagen or abdominal wall defects *via* the TGF-β/PI3K/Akt signaling pathway, resulting in inguinal hernias.

In this article we expect to discover some of the potential genetic markers and related drugs involved in inguinal hernia formation. Bendavid suggests that pathological changes in collagen underlie the pathogenesis of hernias and that the MMP family may play a role in hernia pathogenesis by degrading collagen [52, 68]. MMP-2 is abnormally elevated at both mRNA and protein levels in the serum and fascial tissues of patients with abdominal wall hernias. *In vitro* studies have shown that epigallocatechin gallate (EGCG) reduces MMP-1 and MMP-13 expression levels and inhibits proteoglycan and collagen degeneration in human cartilage [69]. Further preclinical and clinical research is required to ascertain whether inhibiting MMPs with EGCG could serve as a novel targeted intervention in the pathogenesis of inguinal hernias. Rosuvastatin reduces collagen content by inhibiting the expression of MMP-2 and MMP-9 in myocardial tissue [70]. Besides, go enrichment analysis pathway results for hub genes IGF1, IL6, MMP2, TNF, and IL10 showed close association with regulation of muscle cell proliferation, smooth muscle cell proliferation, and muscle cell proliferation.

Rosuvastatin effectively inhibits the expression of IL-1β, IL-6 and TNF mRNA and protein in middle cerebral artery smooth muscle cells [71], consequently decreasing the post-ischemia-reperfusion inflammatory response. Numerous studies have demonstrated that inguinal hernias are associated with muscle tissue conditions. On the one hand, there is reason to believe that these genes could be investigated further as potential inguinal hernia targets. Complementing the previous point, we also propose an alternative hypothesis regarding whether the inguinal hernia is a systemic disease: a local disease and novel drug indications for the existing drug (rosuvastatin). Currently, the relationship between chembl1945287, fludeoxyglucose, puromycin, adalimumab, vonapanitase, antibiotics, baclofen, rilotumumab, ficlatuzumab, clugine, fontolizumab, dusigitumab, xentuzemab, sch-708980, siltuximab, rebimastat, obovatal, NIS-793, golimumab, ranibizumab, caplacizumab, egaptivon pegol and there are no reports of inguinal hernia in the current literature. Future research endeavors could delve deeper into exploring the intricate relationship between these substances and inguinal hernia, potentially uncovering more comprehensive insights.

Through using the Cytoscape software, the MCODE analysis generated 37 hub genes. According to current research, no studies have been conducted on the effect of other hub genes on inguinal hernia.

This is the first study to explore the causal association between hub genes' cis-expression quantitative trait loci (eQTLs) levels and inguinal hernia risk by a two-sample MR analysis based on a large amount of GWAS data of hub genes (exposure) and inguinal hernia (outcome). This MR study showed that nine genes that may be connected with inguinal hernia. These genetic candidates encompass POMC, CD40LG, TFRC, VWF, LOX, IGF2, BRCA1, TNF, and HGF in the plasma, shedding light on potential molecular underpinnings of this condition. Moreover, albumin (ALB) was ranked the highest connectivity gene in the PPI network, as well as possessing higher interaction scores in drug-gene interactions. Serum ALB levels might be causally associated with an increased risk of inguinal hernia. In the context of this research, Mendelian randomization (MR) shares a fundamental similarity with prospective randomized controlled trials (RCTs). However, MR offers the advantage of reducing systematic biases that can impact the outcomes of conventional observational studies. These biases include factors like confounding variables and reverse causality. Furthermore, the high precision of genotyping plays a crucial role in mitigating regression dilution stemming from detection errors. To ensure that the selected single-nucleotide polymorphisms (SNPs) remained unrelated to any confounding factors influencing the association between ALB and inguinal hernia, we specifically included participants from European populations. Lastly, to validate the robustness of our findings, we conducted an MR–Egger regression test, which yielded no evidence of directional pleiotropy. In summary, this study suggests that ALB holds promise as a potential therapeutic target for addressing inguinal hernia.

## CONCLUSION

In summary, our study has successfully identified 37 potential hub genes associated with inguinal hernia. These genes have exhibited significant enrichment in critical pathways, including HIF-1, PI3K-Akt, MAPK, and AGE pathways. Furthermore, our research has unveiled a collection of 30 drugs that hold promise for guiding future therapeutic strategies for individuals with inguinal hernia.

These hub genes were reverse-validated potential genes by Mendelian randomization. The lack of experimental validation is a limitation of this study, and further experimental studies are needed to validate these results. The identified drugs could be potential candidates for the treatment of patients with inguinal hernia.

## Figures and Tables

**Fig. (6):**
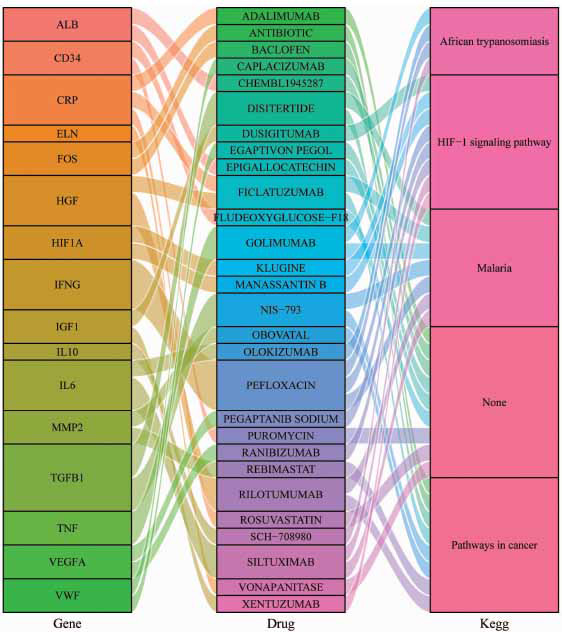
Sankey diagram display the essential connections among drugs, genes, and pathways. Drug-gene interactions were analyzed for 16 genes in module 1. We found 16 genes that target 30 potential existing drugs. In addition, these 16 genes are mainly enriched in 4 KEGG pathways. “None” in the pathway means that the hub gene has no related pathway.

**Table 1:** The top 10 gene ontology (GO) terms enriched among the text mining genes.

**Progress**	**Genes in Query Set**	**Total Genes** **in Genome**	**Corrected** **hypergeometric** ***p* Value**	**Genes**
Positive regulation of gene expression	18	516	2.19E-10	TFRC, TGFB1, BRCA1, CRP, CD34, SRY, IFNG, SOX9, ZMPSTE24, PDGFB, TNF, HIF1A, VEGFA, AR, IL6, LMNA, IGF1, AMH
Negative regulation of apoptotic process	17	529	2.56E-09	ALB, CD38, TFRC, FLNA, HGF, IL10, CD40LG, LRP2, GCG, SOX9, TNF, HIF1A, VEGFA, IL6, MSX2, IGF1, BCL2
Response to activity	7	44	3.20E-09	IL10, GCG, MMP2, TNF, IL6, ZEB1, SLC25A25
Negative regulation of cell population proliferation	16	463	2.84E-09	IL10, COL4A3, NUDT6, IRF6, TGFB1, NF1, SOX9, TNF, AR, IL6, MSX2, GPC3, ZEB1, LMNA, SST, BCL2
Positive regulation of miRNA transcription	7	46	4.44E-09	IL10, FOS, TGFB1, PDGFB, TNF, HIF1A, PRL
Positive regulation of peptidyl-tyrosine phosphorylation	8	96	4.39E-08	HGF, IGF2, TGFB1, GH1, PDGFB, VEGFA, IL6, IGF1
Positive regulation of transcription, DNA-templated	18	734	5.44E-08	CD38, IL10, MAX, FOS, CDKN1C, IRF6, CREBBP, TGFB1, BRCA1, ZFPM2, SRY, SOX9, PDGFB, TNF, HIF1A, AR, IL6, IGF1
Response to xenobiotic stimulus	11	253	1.05E-07	ABCB1, CD38, IL10, FOS, MMP2, TNF, SRD5A1, AMH, LOX, SST, BCL2
Positive regulation of phosphatidylinositol 3-kinase signaling	7	81	2.48E-07	HGF, GH1, SOX9, PDGFB, TNF, VEGFA, IGF1
Regulation of gene expression	12	346	3.08E-07	SHC4, IL10, FOS, NF1, SOX9, COL2A1, HIF1A, AR, NOVA2, IGF1, LOX, BCL2

**Table 2:** Top 10 enriched KEGG pathways assigned to the text mining genes.

**Progress**	**Genes in Query Set**	**Total Genes in Genome**	**Corrected Hypergeometric** ***p* Value**	**Genes**
Malaria	7	50	3.38E-07	HGF, IL10, CD40LG, TGFB1, IFNG, TNF, IL6
EGFR tyrosine kinase inhibitor resistance	8	79	6.07E-07	SHC4, HGF, NF1, PDGFB, VEGFA, IL6, IGF1, BCL2
HIF-1 signalling pathway	9	109	6.67E-07	TFRC, CREBBP, NPPA, IFNG, HIF1A, VEGFA, IL6, IGF1, BCL2
Focal adhesion	10	201	1.55E-05	SHC4, FLNA, HGF, COL4A3, PDGFB, COL2A1, VEGFA, VWF, IGF1, BCL2
African trypanosomiasis	5	36	1.87E-05	IL10, NPPA, IFNG, TNF, IL6
Cytokine-cytokine receptor interaction	12	293	1.47E-05	IL10, CD40LG, AMHR2, TGFB1, GH1, TNFRSF25, IFNG, TNF, TNFRSF1B, PRL, IL6, AMH
Proteoglycans in cancer	10	205	1.84E-05	FLNA, HGF, IGF2, TGFB1, MMP2, TNF, HIF1A, VEGFA, GPC3, IGF1
PI3K-Akt signaling pathway	13	353	1.94E-05	HGF, COL4A3, IGF2, BRCA1, GH1, PDGFB, COL2A1, VEGFA, PRL, IL6, VWF, IGF1, BCL2
Pathways in cancer	16	531	2.34E-05	HGF, MAX, COL4A3, IGF2, FOS, CREBBP, TGFB1, IFNG, MMP2, PDGFB, HIF1A, VEGFA, AR, IL6, IGF1, BCL2

**Table 3:** Hub node genes in the PPI network identified with filtering node degree≥10.

**Progress**	**Genes in Query Set**	**Total Genes in Genome**	**Corrected Hypergeometric *p* Value**	**Genes**
Malaria	7	50	3.38E-07	HGF, IL10, CD40LG, TGFB1, IFNG, TNF, IL6
EGFR tyrosine kinase inhibitor resistance	8	79	6.07E-07	SHC4, HGF, NF1, PDGFB, VEGFA, IL6, IGF1, BCL2
HIF-1 signaling pathway	9	109	6.67E-07	TFRC, CREBBP, NPPA, IFNG, HIF1A, VEGFA, IL6, IGF1, BCL2
Focal adhesion	10	201	1.55E-05	SHC4, FLNA, HGF, COL4A3, PDGFB, COL2A1, VEGFA, VWF, IGF1, BCL2
African trypanosomiasis	5	36	1.87E-05	IL10, NPPA, IFNG, TNF, IL6
Cytokine-cytokine receptor interaction	12	293	1.47E-05	IL10, CD40LG, AMHR2, TGFB1, GH1, TNFRSF25, IFNG, TNF, TNFRSF1B, PRL, IL6, AMH
Proteoglycans in cancer	10	205	1.84E-05	FLNA, HGF, IGF2, TGFB1, MMP2, TNF, HIF1A, VEGFA, GPC3, IGF1
PI3K-Akt signaling pathway	13	353	1.94E-05	HGF, COL4A3, IGF2, BRCA1, GH1, PDGFB, COL2A1, VEGFA, PRL, IL6, VWF, IGF1, BCL2
Pathways in cancer	16	531	2.34E-05	HGF, MAX, COL4A3, IGF2, FOS, CREBBP, TGFB1, IFNG, MMP2, PDGFB, HIF1A, VEGFA, AR, IL6, IGF1, BCL2

**Table 4:** Details of the 30 drugs that potentially target of the 16 hub genes.

**Drug**	**Gene**	**Interaction Type & Directionality**	**Interaction Score**	**PMIDs**
CHEMBL1945287	ALB	N/A	5.89	21632244
EPIGALLOCATECHIN	ALB	N/A	5.89	20598557
FLUDEOXYGLUCOSE-F18	CD34	N/A	5.15	16513610
PUROMYCIN	CD34	N/A	3.86	11973349
ADALIMUMAB	CRP	N/A	2.42	27096233
ROSUVASTATIN	CRP	N/A	3.17	21094359
VONAPANITASE	ELN	N/A	123.66	None found
ANTIBIOTIC	FOS	N/A	1.24	15773551
BACLOFEN	FOS	N/A	1.24	11301212
RILOTUMUMAB	HGF	Antibody	12.88	None found
FICLATUZUMAB	HGF	Antibody	12.88	None found
KLUGINE	HIF1A	N/A	0.91	15974627
MANASSANTIN B	HIF1A	N/A	0.91	15165135
FONTOLIZUMAB	IFNG	Inhibitor	7.27	None found
DUSIGITUMAB	IGF1	Inhibitor	30.91	None found
XENTUZUMAB	IGF1	N/A	15.46	None found
SCH-708980	IL10	Inhibitor	3.64	None found
SILTUXIMAB	IL6	Antagonist	9.89	8823310
OLOKIZUMAB	IL6	Inhibitor	9.89	24641941
REBIMASTAT	MMP2	N/A	5.15	15041713
OBOVATAL	MMP2	N/A	5.15	17428670
DISITERTIDE	TGFB1	N/A	3.34	24393789
NIS-793	TGFB1	N/A	1.67	None found
GOLIMUMAB	TNF	Antibody	4.55	21079302
RANIBIZUMAB	VEGFA	Inhibitor	8.54	18046235
PEGAPTANIB SODIUM	VEGFA	Antagonist	3.25	23953100
CAPLACIZUMAB	VWF	Inhibitor	13.25	None found
EGAPTIVON PEGOL	VWF	N/A	4.42	None found

## Data Availability

The study's original contributions are included in the article; further questions can be directed to the article's corresponding authors.

## LIST OF ABBREVIATIONS

DAVID: Database for Annotation, Visualization and Integrated Discover
DGIDB: Drug-gene Interaction Database
EGCG: Epigallocatechin Gallate
GO: Gene Ontology
IGF1: Insulin-like Growth Factor-1 receptor
KEGG: Kyoto Encyclopedia of Genes and Genomes
MCODE: Molecular Complex Detection
MMPs: Matrix Metalloproteinases
SFKs: Src Family of Kinases
