# Supplementary material for: Functional Investigation and Two-sample Mendelian Randomization Study of Inguinal Hernia Hub Genes Obtained by Bioinformatics Analysis
Source: Curr Comput Aided Drug Des. 2024 Apr 5;21(6):909–24. doi: 10.2174/0115734099282407240325054745 (PMC12824866; doi:10.2174/0115734099282407240325054745)
Supplement: Supplementary file 1 [file CCADD-21-6-909_SD1.zip › CCADD-21-6-909_SD1/CCADD-21-6-12-Supply.pdf]

SUPPLEMENTARY MATERIAL

Functional Investigation and Two-sample Mendelian Randomization Study of Inguinal Hernia Hub Genes Obtained by Bioinformatics Analysis

De Kun Lu[1], Zheng Chang Guo[2], Jia Jia Zhang[3], Xin Yu[3] and Zong Yao Zhang[1,*]

[1]Department of General Surgery, The First Affiliated Hospital of Anhui University of Science and Technology, No.203 Huai Bin Road, Tian Jia'an District, Huainan, 232007, China; [2]Department of General Surgery, Zhenjiang First People's Hospital, No.8 Electricity Road, Run Zhou District, Zhenjiang, China; [3]Department of General Surgery, The First Affiliated Hospital of Anhui Medical University, No. 218 Jixi Road, 230022, Hefei, China
